# 57963 The Impact of Asian American Perceived Discrimination on Health Utilization

**DOI:** 10.1017/cts.2021.623

**Published:** 2021-03-31

**Authors:** Thomas Le, Gilbert Gee, Lorraine Dean, Hee-Soon Juon, Som Saha

**Affiliations:** 1 Johns Hopkins Bloomberg School of Public Health; 2 UCLA Fielding School of Public Health; 3 Thomas Jefferson University

## Abstract

ABSTRACT IMPACT: Understanding how perceived discrimination affects Asian Americans can help stakeholders target subgroups that are at highest risk of discrimination-related behaviors and design culturally appropriate interventions to ensure equitable access to healthcare. OBJECTIVES/GOALS: The COVID-19 pandemic has exposed longstanding anti-Asian racism in the US. Yet, effects of discrimination on Asian American health are unknown, partly because diverse Asian American populations are analyzed in aggregate. We aim to understand how perceived discrimination affects healthcare utilization among different Asian American subgroups. METHODS/STUDY POPULATION: We examine the association of perceived discrimination with healthcare utilization using the California Health Interview Survey (CHIS). In the CHIS, respondents reported whether they would’ve gotten better medical care if they belonged to a different race. We examine the association between these responses and physician visits within the past year, in the survey years 2003, 2004 and 2016-2017. We adjust for covariates based on the Andersen Health Behavior model. Subsequent modeling examines potential mediating and moderating factors such as limited English proficiency, immigration status, income, and survey year. Asian American subgroups analyzed include Asian Indian, Korean, Chinese, Filipino, Vietnamese, Japanese, and other Asian. RESULTS/ANTICIPATED RESULTS: Results will highlight how perceived discrimination incentivizes or disincentivizes certain Asian subgroups to utilize healthcare. Asian American subgroups have differing and diverse experiences with discrimination due to their historical and cultural differences; results will elucidate how discrimination affects these subgroups. Results will be compared to non-Hispanic Whites, who represent the racial group least likely to experience discrimination in the US. Mediation and moderation analysis will help understand how traditionally cited factors for healthcare utilization interact with perceived discrimination on Asian Americans. DISCUSSION/SIGNIFICANCE OF FINDINGS: Asian American subgroups are understudied, despite Asian Americans being one of the fastest growing racial groups in the US. Understanding how perceived discrimination affects Asian Americans can help stakeholders target subgroups that are at highest risk of discrimination-related behaviors and design culturally appropriate interventions.

